# Molecular and morphological characterization of the alfalfa cyst nematode, *Heterodera medicaginis*, from Utah

**DOI:** 10.21307/jofnem-2020-015

**Published:** 2020-03-17

**Authors:** Zafar A. Handoo, Andrea M. Skantar, Saad L. Hafez, Mihail R. Kantor, Maria N. Hult, Stephen A. Rogers

**Affiliations:** 1 Mycology and Nematology Genetic Diversity and Biology Laboratory, USDA, ARS, Northeast Area, Beltsville, MD, 20705; 2 University of Idaho, Parma, ID, 83660

**Keywords:** *Heterodera medicaginis*, Alfalfa, Utah, Nematodes.

## Abstract

In the spring of 2019, a cyst nematode was discovered from soil samples collected from an alfalfa field in Millard County, Utah. The soil samples were submitted to one of us (SH), who extracted the nematode cysts and sent them to the USDA-ARS, Mycology and Nematology Genetic Diversity and Biology Laboratory (MNGDBL), Beltsville, MD for morphological and molecular identification. Cysts and living nematode juveniles (J2) recovered from the cysts were examined morphologically and molecularly for species identification which indicated that the specimens were *Heterodera medicaginis.* This represents the first record of *H. medicaginis* in Utah and the second report of this nematode in North America.

The alfalfa cyst nematode, *Heterodera medicaginis* Kirjanova in the study of Kirjanova and Krall (1971) was originally described from the USSR in 1971 and re-described by Gerber and Maas (1982). The distribution of *H. medicaginis* is mostly Europe (Russia and Ukraine) and Asia (Kazakhstan and Uzbekistan) (Subbotin et al., 2010). This cyst nematode species can cause up to 46% green matter losses in lucerne under arid conditions (Artokhnica, 1984; Subbotin et al., 2010). More recently, the first report of the alfalfa cyst nematode in North America was reported in Kansas by Powers et al. (2019). Previously, unpublished records of this cyst species reproducing on alfalfa indicated the presence of this nematode in the Great Plains states of Kansas and Montana (Powers et al., 2019).

The soil sample was collected from Delta, Utah (Millard county) and sent to University of Idaho for initial evaluation, after which it was submitted to USDA-ARS in Beltsville, MD for morphological and molecular identification.

## Material and methods

Various stages: cysts, second-stage juveniles (J2), and eggs were sent to MNGDBL, Beltsville, MD from the University of Idaho. Juveniles used for morphological observations were separated from soil by sieving and Baermann funnel extraction. Juveniles were fixed in 3% formaldehyde and processed to glycerin by the formalin glycerin method (Hooper, 1970; Golden, 1990). Females and some cysts were typically removed from roots after fixation for 12 hr in 3% formaldehyde solution. Photomicrographs of cyst vulval cones, females, and J2 were made with an automatic 35-mm camera attached to a compound microscope having an interference contrast system. Roots and whole cysts were photographed under a dissecting microscope, and light microscopic images of fixed nematodes were taken on a Nikon Eclipse Ni compound microscope using a Nikon DS-Ri2 camera. Measurements were made with an ocular micrometer on a Leica WILD MPS48 Leitz DMRB compound microscope. All measurements are in micrometers, unless otherwise stated.

Living nematode juveniles (J2) recovered from cysts were examined morphologically and molecularly for species identification.

The ITS 1&2 rDNA region was amplified with primers TW81 and AB28 (Skantar et al., 2012), producing a PCR amplicon of 985 bp. The PCR product was cleaned with the Monarch DNA Gel Extraction Kit (NEB, Ipswitch, MA) and then cloned using the Strataclone PCR Cloning Kit (Agilent, Santa Clara, CA). Cloned plasmid DNA was prepared with the Monarch Plasmid Miniprep Kit (NEB) and sequenced by Genewiz, Inc. The ITS rDNA sequence was assigned GenBank accession number MN308440. Hsp90 primers U831 [5′-AA(T/C)AA(A/G)AC(A/C) AAGCC(A/C/G/T)T(T/C)TGGAC-3′] and L1110 [5′-TC(A/G)CA(A/G)TT(G/A/C) TCCATGAT(A/G)AA(G/A/C)AC-3′] (Skantar and Carta 2004) were used to amplify a PCR product of 437 bp, which was cloned and sequenced as described above. Six clones containing the Hsp90 fragment were sequenced and submitted to GenBank under accession numbers MN311173-MN311178. Mitochondrial cytochrome oxidase I (COI) was amplified with primers Het-Cox1F and Het-Cox1R as described in the study of Subbotin et al. (2017). PCR amplicons of 430 bp were cleaned and sequenced directly with the same primers. The three identical sequences were submitted to GenBank under the accession number MN311179. DNA sequences were analyzed by BlastN. Evaluations of intraspecific and interspecific variation were conducted using sequence alignment algorithms within Geneious.

## Description

### Measurements

**Figure 1:**
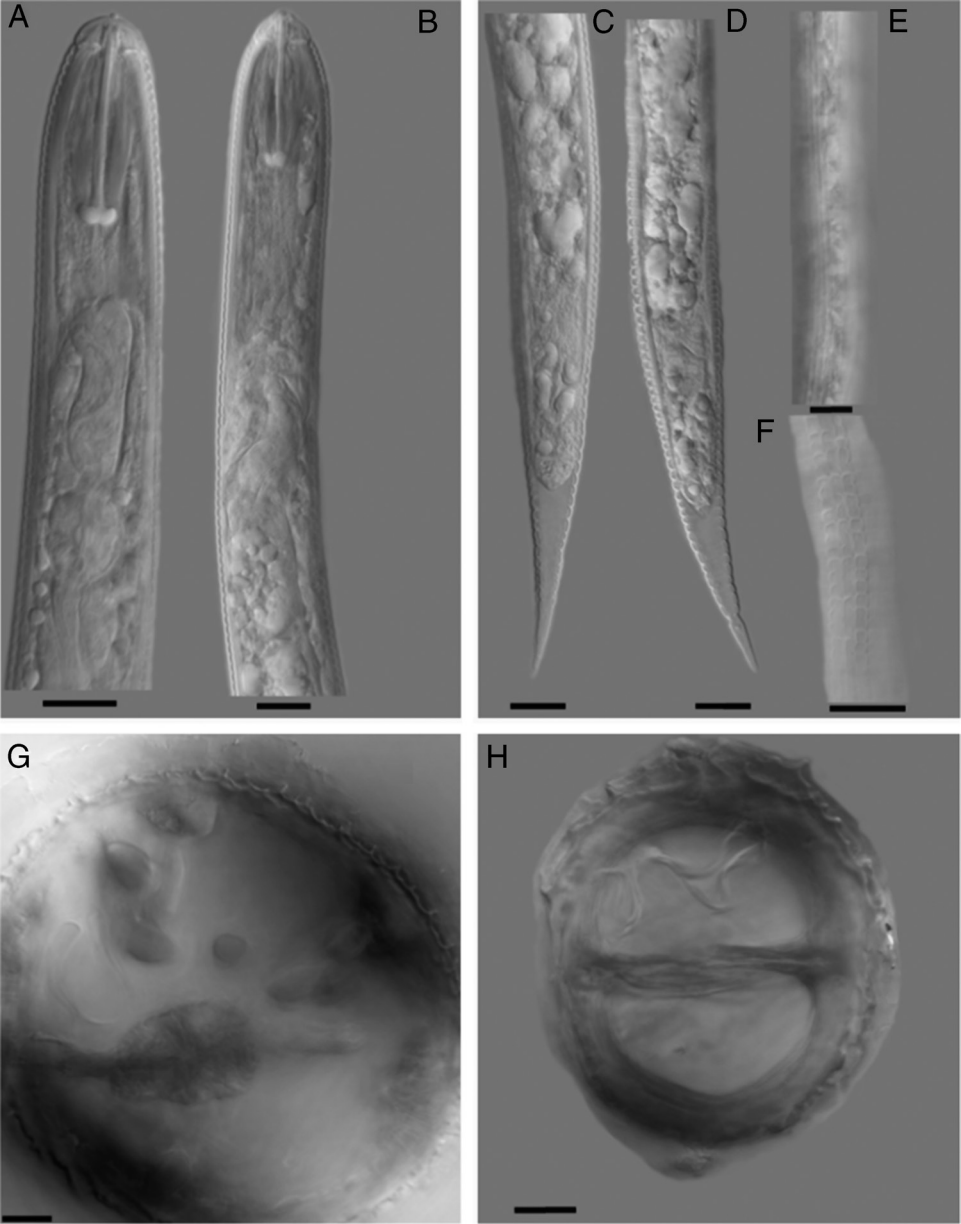
Photomicrographs of second-stage juveniles (A-F) and vulva cones (G and H) of *Heterodera medicaginis*. A-B heads; C-D tails; E-F lateral field; G-H cone mounts, G showing the bullae and H showing the underbridge. The scale bar=10 μm.

Measurements of second-stage juveniles (*n* = 15) included length of body (range = 435-510 μm, mean=484 μm), stylet well developed (23.5-26.0 μm, 24.96 μm) with anchor-shaped basal knobs, long basal bulb (75 μm) with three nuclei, tail (50-65.0 μm, 55.8 μm), and hyaline tail terminus (25-35 μm, 30.3 μm). The lateral field had four distinct lines with few specimens having an aerolated lateral field (Fig. 1F), which to our knowledge has not been reported so far. The cysts (*n* = 5) were dark brown in color, lemon shaped, ambifenestrate with heavy bullae, underbridge and having a zig-zag cyst wall cuticular pattern (Fig. 1G,H); punctuations often present in terminal area of cyst. Measurements of the cysts included: cyst length including the neck (range = 327-445 μm, mean = 400 μm, SD = 48.9 μm), cyst width (160-232 μm, 208.4 μm, and 29 μm), neck length (40-50 μm, 43 μm, and 4.4 μm), and neck width (34-40 μm, 35.8 μm, and 2.3 μm). Observations of morphological characters critical for identification (Fig. 1A-F) including shapes of the tail, tail terminus, stylet knobs of the J2’s as well as the morphometrics of cysts indicated that the specimens were *Heterodera medicaginis*.

### Molecular analysis

DNA sequences from the Utah population were compared with those previously obtained from Kansas and Montana specimens (Powers et al., 2019) and Russia (Subbotin et al., 2010). The ITS rDNA sequence was > 99.1% identical to the *H. medicaginis* sequence from Russia (AF274391) and several from Kansas (accession numbers MK093180-MK093186). The mitochondrial Cox1 sequence shared > 99.51% identity to several *H. medicaginis* sequences from Kansas (MK093177-MK093179; MK093168-MK093172). Six cloned haplotypes of the Hsp90 short fragment was obtained from the Utah population. These clone sequences varied from 0 to 4 bp among each other and between 3 and 8 bp from those from Kansas (MH798843-MH798846). Based upon the unambiguous similarity of all examined DNA markers with those previously reported for the species, we conclude these populations are from *H. medicaginis.*

Based upon this collective morphological and molecular data, we identify this isolate as *Heterodera medicaginis.* To our knowledge this is the first report of the alfalfa cyst nematode in Utah.
